# Ultrasound-Guided Perineural Corticosteroid Injection and Dextrose Hydrodissection for Carpal Tunnel Syndrome: Current Evidence and Clinical Considerations

**DOI:** 10.7759/cureus.112663

**Published:** 2026-07-14

**Authors:** Michael J Thornton, Nicholas J Thornton, Simron Gill, Josette Hartnett, Bram Newman

**Affiliations:** 1 Physical Medicine and Rehabilitation, Burke Rehabilitation Hospital, White Plains, USA; 2 Sports Medicine, Burke Rehabilitation Hospital, White Plains, USA; 3 Research, Burke Rehabilitation Hospital, White Plains, USA

**Keywords:** carpal tunnel syndrome, corticosteroid injection, d5w hydrodissection, entrapment neuropathy, median nerve, musculoskeletal ultrasound, perineural injection, peripheral nerve hydrodissection, regenerative injection, ultrasound-guided injection

## Abstract

Carpal tunnel syndrome (CTS) is the most common entrapment neuropathy and represents a substantial source of pain, disability, and healthcare expenditure. Ultrasound-guided perineural corticosteroid injection (CSI) remains the most established injectable treatment for CTS; however, hydrodissection with 5% dextrose in water (D5W) has emerged as a promising alternative that may address both inflammatory and mechanical contributors to median nerve entrapment. Our objective is to review the current evidence comparing ultrasound-guided perineural CSI and D5W hydrodissection for the nonoperative treatment of CTS and to discuss clinical considerations regarding their use. A narrative review of the literature was performed using PubMed/MEDLINE and Google Scholar through January 2026. Search terms included “carpal tunnel syndrome,” “median nerve,” “corticosteroid injection,” “hydrodissection,” “perineural injection,” “dextrose,” and “D5W.” Priority was given to randomized controlled trials, systematic reviews, meta-analyses, clinical practice guidelines, and prospective cohort studies evaluating CSI, D5W hydrodissection, or direct comparisons between these interventions. CSI remains supported by a robust body of evidence demonstrating reliable short-term improvements in pain, paresthesias, and functional outcomes in patients with mild-to-moderate CTS. D5W hydrodissection has demonstrated favorable clinical and sonographic outcomes, with several studies reporting sustained symptom improvement beyond the time frame typically observed with CSI. Direct comparative studies suggest that D5W hydrodissection may provide longer-lasting symptom relief in some patients; however, current evidence remains limited by small sample sizes, heterogeneity in hydrodissection techniques, and a lack of large multicenter randomized controlled trials. Both ultrasound-guided perineural CSI and D5W hydrodissection are effective nonoperative treatment options for CTS. CSI remains the current first-line injectable therapy because of its extensive evidence base and established efficacy. D5W hydrodissection represents a promising alternative that may provide longer-lasting symptom improvement while avoiding corticosteroid-related adverse effects. Additional high-quality comparative studies are needed to further define optimal patient selection and the long-term role of D5W hydrodissection in CTS management.

## Introduction and background

Carpal tunnel syndrome (CTS) is the most common entrapment neuropathy, representing approximately 90% of all entrapment neuropathies [1]. CTS has a lifetime prevalence of up to 10% and occurs more frequently in women, with an estimated female-to-male ratio of 3:1 [2]. Patients typically present with pain, numbness, and tingling in the median nerve distribution that commonly worsen at night [1]. CTS also imposes a substantial economic burden, with an estimated annual cost of $2.7-$4.8 billion in the United States, largely attributable to lost productivity and missed work [3]. Patients with CTS miss an average of 28 workdays per episode, often resulting in persistent earnings losses [4].

Nonoperative management is generally recommended as the initial treatment for patients with mild-to-moderate CTS, particularly those without thenar muscle atrophy, significant motor weakness, or severe electrodiagnostic abnormalities. Conservative treatment aims to reduce median nerve inflammation, improve nerve gliding, preserve hand function, and delay or avoid surgical decompression. Common first-line therapies include wrist splinting, activity modification, physical therapy, non-steroidal anti-inflammatory drugs, and an increasing use of ultrasound-guided interventions [5]. Ultrasound-guided perineural corticosteroid injection (CSI) remains the most established injectable intervention for CTS and consistently provides clinically meaningful short-term improvements in pain and function [6].

Hydrodissection has emerged as an alternative ultrasound-guided technique in which fluid is injected around the median nerve to mechanically separate it from surrounding tissues, restore a perineural tissue plane, and improve nerve mobility. This approach differs from a conventional perineural injection because its objective is not simply medication delivery but mechanical release of potential adhesions and restoration of nerve gliding. Platelet-rich plasma injection and hydrodissection using various injectates have gained increasing interest; however, evidence remains heterogeneous, and procedural techniques have not been fully standardized [5]. Among the currently available hydrodissection injectates, 5% dextrose in water (D5W) is one of the most extensively studied and has demonstrated favorable clinical outcomes in several randomized trials and systematic reviews [7]. Nevertheless, additional high-quality comparative studies are needed before its role relative to CSI can be fully established.

The purpose of this narrative review is to evaluate the current evidence comparing ultrasound-guided perineural CSI and D5W hydrodissection for the nonoperative treatment of CTS. As hydrodissection continues to gain popularity among clinicians performing ultrasound-guided interventions, understanding its relative efficacy, safety profile, procedural considerations, and durability of symptom relief compared with CSI has become increasingly important. D5W hydrodissection may represent an attractive option for selected patients who wish to avoid corticosteroid exposure, including individuals with poorly controlled diabetes mellitus, corticosteroid intolerance, or concerns regarding corticosteroid-related adverse effects [8]. In comparison with corticosteroids, D5W has minimal systemic adverse effects and can be repeated without cumulative steroid exposure. Electrodiagnostic studies, including nerve conduction studies and electromyography, remain the reference standard for confirming median neuropathy and identifying axonal injury, particularly in patients with severe or atypical presentations. Patients with severe CTS, including those with thenar atrophy, persistent sensory deficits, significant motor weakness, or severe electrodiagnostic abnormalities despite conservative management, should generally be referred for consideration of surgical decompression rather than injection therapy [9].

## Review

Methodology

A narrative review of the literature was performed to evaluate the current evidence regarding ultrasound-guided perineural CSI and hydrodissection with D5W for the treatment of CTS. The literature search was conducted between January 1 and January 31, 2026, using PubMed/MEDLINE and Google Scholar. Search terms included combinations of “carpal tunnel syndrome,” “median nerve,” “corticosteroid injection,” “steroid injection,” “hydrodissection,” “perineural injection,” “dextrose,” and “D5W.”

Only English-language studies involving human subjects were included. Priority was given to randomized controlled trials, systematic reviews, meta-analyses, clinical practice guidelines, prospective cohort studies, and comparative clinical studies evaluating CSI, D5W hydrodissection, or direct comparisons between these interventions. Technique papers and other clinically relevant publications were also considered when they provided important information regarding ultrasound-guided injection or hydrodissection procedures. Additional references were identified through manual review of bibliographies from relevant publications. Editorials, conference abstracts, and studies not directly addressing the clinical management of CTS were excluded. Articles were selected based on their relevance to the objectives of this focused narrative review and their contribution to the current understanding of ultrasound-guided injectable therapies for CTS.

Because this was a focused narrative review rather than a systematic review, formal study screening, risk-of-bias assessment, and Preferred Reporting Items for Systematic Reviews and Meta-Analyses (PRISMA) methodology were not performed. The objective was to synthesize the current evidence and discuss practical clinical considerations regarding ultrasound-guided CSI and D5W hydrodissection.

Owing to substantial heterogeneity in study design, hydrodissection techniques, injectate volumes, treatment protocols, outcome measures, CTS severity, and follow-up duration, a formal meta-analysis was not performed.

Pathophysiology of carpal tunnel syndrome

CTS is the most common entrapment neuropathy and occurs when the median nerve is compressed as it passes through the carpal tunnel at the wrist. The carpal tunnel is an osteofibrous canal located on the palmar side of the wrist. Its floor and walls are formed by the carpal bones, while its roof is formed by the transverse carpal ligament (flexor retinaculum). The tunnel contains the median nerve and nine flexor tendons that control finger and thumb movement. Because this space is limited, any increase in tissue volume or pressure within the tunnel can compress the median nerve. Common consequences include numbness, tingling, pain, and weakness in the thumb, index, middle, and radial half of the ring finger. When pressure within the tunnel rises, the median nerve may develop impaired venous outflow, reduced intraneural blood flow, edema, and ischemia. With persistent compression, these changes may progress to segmental demyelination and, in more severe or chronic cases, axonal injury [1,10].

The pathophysiology of CTS is multifactorial and includes mechanical, vascular, and biological contributors. Increased carpal tunnel pressure can impair nerve conduction and produce the classic symptoms of nocturnal paresthesias, numbness, pain, and weakness in the median nerve distribution. In addition to direct compression, histologic and biomechanical studies have demonstrated thickening and fibrosis of the subsynovial connective tissue surrounding the flexor tendons. This subsynovial connective tissue normally allows smooth tendon and nerve movement during wrist and finger motion. In CTS, noninflammatory fibrosis and reduced tissue compliance may limit median nerve mobility and contribute to chronic entrapment [10,11].

These mechanisms provide the rationale for injection-based therapies. CSI is directed primarily at the inflammatory and edematous components of CTS. By reducing synovial swelling, local inflammatory signaling, and perineural edema, corticosteroids may decrease pressure within the carpal tunnel and improve median nerve symptoms. This mechanism explains why CSI often provides relatively rapid symptom relief, particularly in mild-to-moderate CTS. However, CSI does not directly address fixed mechanical compression, subsynovial fibrosis, or impaired nerve gliding. This may help explain why benefits are often temporary and why recurrence can occur over time [5,6,9,12].

Hydrodissection has emerged as an ultrasound-guided technique intended to address both mechanical and biochemical contributors to nerve entrapment. During hydrodissection, a fluid injectate is delivered around the median nerve under ultrasound visualization to separate the nerve from surrounding structures, including the flexor retinaculum, flexor tendons, and subsynovial connective tissue. Various injectates have been used for this purpose, including normal saline, local anesthetics, corticosteroid-containing solutions, platelet-rich plasma, and D5W. This mechanical separation may reduce perineural adhesions, improve median nerve movement, and restore a more favorable gliding environment within the carpal tunnel. Unlike a low-volume CSI, hydrodissection typically uses a higher injectate volume, generally ranging from approximately 5 to 15 mL, to create a visible tissue plane around the nerve [2,7].

Although hydrodissection is frequently discussed as a single intervention, considerable procedural variability exists among published studies. Most investigators perform the procedure using an in-plane ultrasound-guided approach to allow continuous visualization of the needle tip and controlled separation of the median nerve from surrounding tissues. However, differences remain regarding needle trajectory, target tissue planes, superficial versus deep dissection, the degree of circumferential median nerve release, injectate volume, and procedural endpoint. These technical variations may influence clinical outcomes independently of injectate selection and likely contribute to the heterogeneity observed across published studies [2,7].

D5W currently has one of the largest supporting bodies of evidence among hydrodissection injectates and has emerged as one of the most extensively studied options based on accumulating clinical data demonstrating favorable improvements in symptoms and functional outcomes in CTS [2,7,13,14]. In addition to its mechanical effects, D5W may provide therapeutic benefits beyond simple tissue separation. Proposed mechanisms include modulation of neurogenic inflammation, reduction of peripheral nociceptive signaling, and improvement of the perineural environment. Although the precise biochemical mechanisms remain incompletely understood, D5W has gained increasing attention because it appears to offer both mechanical and biologic effects without the potential adverse effects associated with corticosteroids [2,7,13,14].

Thus, CSI and D5W hydrodissection are based on overlapping but distinct therapeutic rationales. CSI primarily targets inflammation and edema, whereas D5W hydrodissection is intended to mechanically separate the median nerve from surrounding tissues while potentially providing additional biologic effects. This distinction is clinically important because CTS likely exists on a spectrum, with some patients having predominantly inflammatory and reversible compression, while others may have more chronic fibrotic or adhesive changes. Understanding these mechanisms helps explain why CSI remains an established first-line injection therapy, while D5W hydrodissection has emerged as a viable alternative and one of the most extensively studied hydrodissection injectates for CTS [2,5,7,9,12-14].

**Figure 1:**
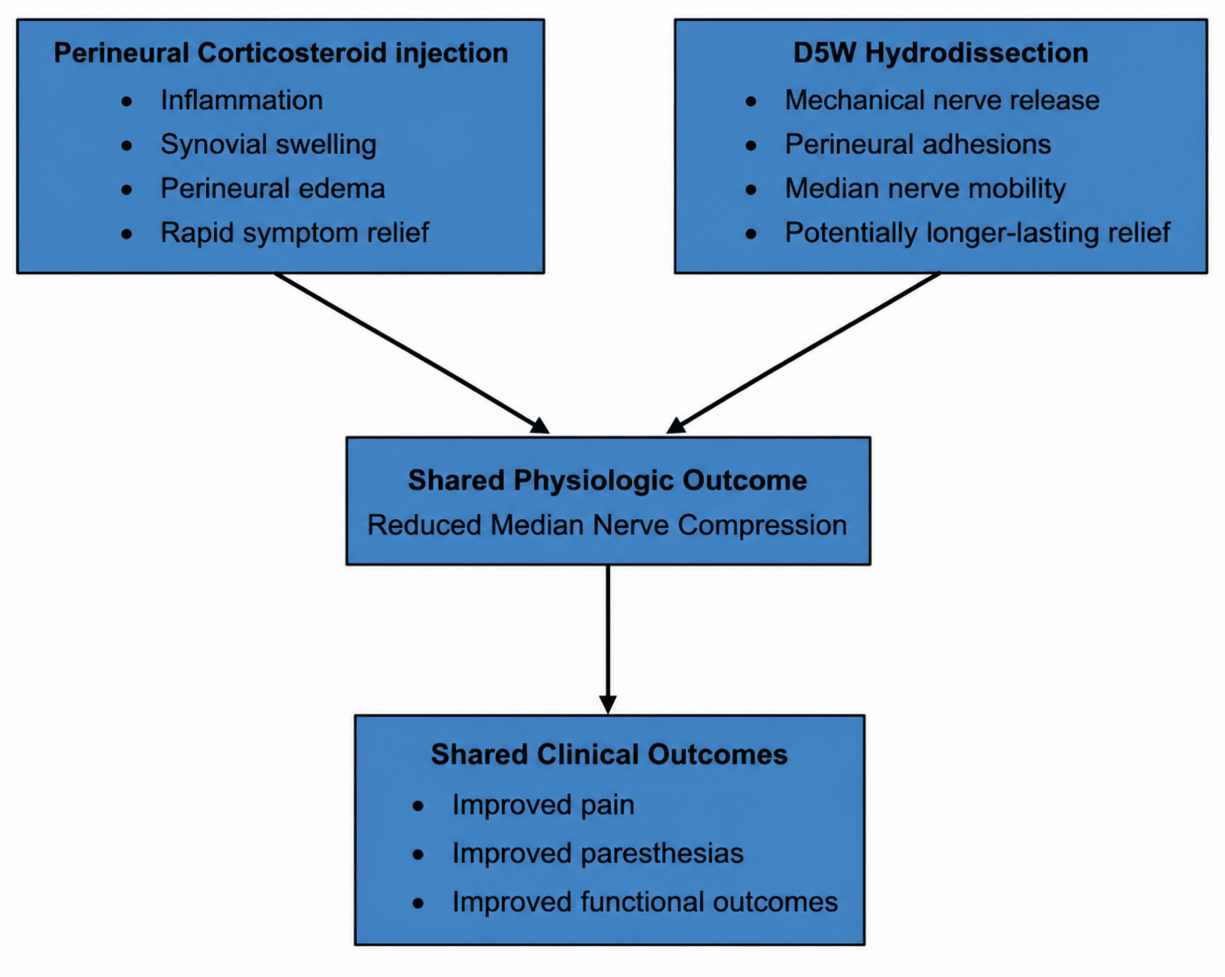
Comparison of the proposed mechanisms of ultrasound-guided perineural corticosteroid injection and D5W hydrodissection in carpal tunnel syndrome. Perineural corticosteroid injection primarily improves symptoms through reduction of local inflammation, synovial swelling, and perineural edema within the carpal tunnel, resulting in decreased median nerve compression and rapid symptom relief. In contrast, D5W hydrodissection is proposed to improve symptoms through mechanical separation of the median nerve from surrounding tissues, reduction of perineural adhesions, restoration of nerve mobility, and potential modulation of neurogenic inflammation. Although the mechanisms differ, both interventions ultimately aim to reduce median nerve compression and improve pain, paresthesias, and functional outcomes in patients with carpal tunnel syndrome. D5W = 5% dextrose in water

The proposed mechanisms of action and shared clinical outcomes of ultrasound-guided perineural CSI and D5W hydrodissection are illustrated in Figure 1.

Comparative evidence between corticosteroid injection and hydrodissection

Supporting Data and Efficacy of Corticosteroid Injection

CSI is the most extensively studied injectable treatment for CTS and remains the current standard of care for patients with mild-to-moderate disease who have failed initial conservative management. The rationale for CSI is based on its ability to reduce local inflammation, synovial swelling, and perineural edema within the carpal tunnel, thereby decreasing pressure on the median nerve and improving symptoms [5,6,12].

Numerous randomized controlled trials and systematic reviews have demonstrated that CSI provides significant short-term improvements in pain, paresthesias, nocturnal symptoms, and functional status compared with placebo and other conservative treatment modalities [6]. Patients frequently experience symptom relief within days to weeks following injection, making corticosteroids an attractive option for individuals seeking rapid improvement or wishing to postpone surgical intervention. Local CSI provides greater symptom relief than placebo and remains an effective nonoperative treatment option for CTS [6].

Although corticosteroid injection is frequently discussed as a single intervention, procedural protocols vary among studies. Triamcinolone and methylprednisolone are the most commonly investigated corticosteroid preparations, with differences in dose and injectate composition reported throughout the literature. Ultrasound-guided CSI typically utilizes approximately 1-3 mL of injectate and is often combined with a small volume of local anesthetic to improve procedural comfort. While local anesthetics are widely used in clinical practice, some authors have questioned their routine use because of theoretical concerns regarding local anesthetic neurotoxicity and possible alteration of injectate pH, although clinical evidence supporting these concerns remains limited [7,15,16].

Ultrasound guidance has further improved the precision and safety of CSI by allowing direct visualization of the median nerve and surrounding structures. Systematic reviews have demonstrated that ultrasound-guided injections may provide superior clinical outcomes, improved procedural accuracy, and reduced risk of inadvertent injury compared with landmark-guided techniques [15,16].

Despite their proven efficacy, CSIs have limitations. Although many patients experience meaningful improvement during the first several months following treatment, symptom recurrence is common and long-term efficacy remains less predictable [5,6]. This limitation likely reflects the fact that corticosteroids primarily address inflammation and edema without correcting the mechanical factors that contribute to median nerve compression. Consequently, some patients ultimately require repeat injections or surgical decompression [9].

Overall, CSI remains an effective, accessible, and evidence-supported treatment option for CTS. Its predictable short-term efficacy, favorable safety profile, and widespread availability have established it as the benchmark against which emerging injectable therapies are compared [5,6,9,12].

Supporting Data and Efficacy of 5% Dextrose in Water Hydrodissection

Hydrodissection has emerged as an increasingly utilized ultrasound-guided intervention for CTS. Unlike CSI, which primarily targets inflammation, hydrodissection aims to mechanically separate the median nerve from surrounding structures while simultaneously delivering a therapeutic injectate. Among the various injectates that have been studied, D5W is among the most extensively investigated [2,7,13,14].

Clinical studies have consistently demonstrated that D5W hydrodissection improves pain, paresthesias, symptom severity, and functional status in patients with CTS. Improvements have been reported using commonly utilized outcome measures, including the Visual Analog Scale and the Boston Carpal Tunnel Questionnaire [2,13,14]. In a prospective randomized double-blind trial, Wu et al. demonstrated that perineural injection of D5W produced significant improvements in pain and functional outcomes that persisted through six months of follow-up [13]. Subsequent studies have reported similar findings and suggest that symptom relief may be maintained beyond the time frame typically observed with CSI alone [17,18].

Published hydrodissection protocols vary considerably. Most studies employ an in-plane ultrasound-guided approach and utilize approximately 5-15 mL of D5W to achieve visible separation of the median nerve from surrounding tissues, although higher-volume techniques have also been described. Differences in needle trajectory, target tissue planes, superficial versus deep hydrodissection, degree of circumferential median nerve release, and number of treatment sessions remain among the principal sources of heterogeneity across published studies. These procedural differences may influence clinical outcomes independently of injectate selection and represent important areas for future standardization [2,7].

In addition to clinical improvement, several studies have demonstrated favorable sonographic changes following D5W hydrodissection, including reductions in median nerve cross-sectional area and improved nerve mobility [2]. These findings support the hypothesis that mechanical separation of the median nerve from surrounding tissues may contribute to symptom improvement by restoring nerve excursion and reducing perineural adhesions.

D5W hydrodissection may be particularly attractive to patients who wish to avoid corticosteroid exposure or who have relative contraindications to steroid administration, including poorly controlled diabetes mellitus. Unlike corticosteroids, D5W does not carry risks of transient hyperglycemia, skin hypopigmentation, or subcutaneous fat atrophy. No clinically significant localized tissue irritation has been consistently reported following perineural D5W injection, and the small amount of dextrose administered does not produce clinically meaningful changes in systemic glucose concentrations. Studies evaluating D5W hydrodissection have also demonstrated a favorable overall safety profile with minimal adverse events [2,7].

Although D5W currently has the strongest clinical evidence among hydrodissection injectates, other biologic injectates have also been investigated. Platelet-rich plasma has demonstrated encouraging early clinical outcomes, whereas therapies such as platelet-poor plasma, alpha-2 macroglobulin, and stem cell-based approaches remain investigational and require substantially more evidence before their role in CTS management can be defined. Because the objective of this review is to compare CSI with D5W hydrodissection, these emerging therapies are not discussed in detail [5,14].

Key clinical studies evaluating D5W hydrodissection and comparative outcomes in CTS are summarized in Table 1. As shown in Table 1, published studies differ not only in study design but also in injection protocols and follow-up duration, underscoring the current lack of standardized hydrodissection methodology.

**Table 1:** Key clinical studies evaluating D5W hydrodissection and comparative outcomes in carpal tunnel syndrome. D5W = 5% dextrose in water; CTS = carpal tunnel syndrome

Study	Design	Participants	Injection protocol	Comparator	Follow-up	Main findings
Wu et al., 2017 [13]	Prospective randomized double-blind controlled trial	49 patients	Single ultrasound-guided perineural injection of 5 mL D5W	Normal saline	6 months	D5W produced significantly greater improvements in pain, symptom severity, and functional outcomes compared with saline throughout 6 months
Wu et al., 2018 [17]	Randomized double-blind comparative trial	54 wrists (27/group)	Single ultrasound-guided perineural injection of 5 mL D5W	Triamcinolone injection	6 months	Both groups improved initially; D5W demonstrated greater improvements in symptom severity and functional outcomes at 4–6 months
Li et al., 2021 [18]	Retrospective follow-up study	185 patients	One or more ultrasound-guided D5W perineural injections	None	1–3 years	Approximately 88.6% of patients reported sustained long-term symptom improvement following treatment
Chao et al., 2022 [19]	Retrospective study	36 patients	Ultrasound-guided D5W hydrodissection for persistent or recurrent CTS	None	Mean: 33 months	Significant long-term improvements in symptoms and function were observed in patients with persistent or recurrent CTS

Comparison of Corticosteroid Injection and 5% Dextrose in Water Hydrodissection

Both CSI and D5W hydrodissection have demonstrated efficacy in the treatment of CTS, yet they differ in their proposed mechanisms of action, procedural characteristics, and duration of benefit. CSI primarily targets inflammation and edema, whereas D5W hydrodissection seeks to address both inflammatory and mechanical contributors to median nerve entrapment by restoring a perineural tissue plane and improving nerve mobility [2,7]. Although these interventions are often compared as distinct treatment strategies, considerable procedural variability exists within each technique. Differences in corticosteroid preparation, injectate composition, hydrodissection technique, injectate volume, target tissue planes, and procedural endpoints should be considered when interpreting comparative outcomes [2,7,15,16].

A comparison of the mechanisms, procedural characteristics, clinical advantages, limitations, and current roles of these interventions is provided in Table 2.

**Table 2:** Comparison of procedural characteristics, clinical outcomes, and current roles of ultrasound-guided perineural corticosteroid injection and D5W hydrodissection for carpal tunnel syndrome. D5W = 5% dextrose in water; CTS = carpal tunnel syndrome

Characteristic	Corticosteroid injection	D5W hydrodissection
Primary mechanism of action	Reduction of local inflammation, synovial swelling, and perineural edema	Mechanical separation of the median nerve from surrounding tissues with potential neuroregulatory and anti-inflammatory effects
Procedural objective	Deliver corticosteroid around the median nerve to reduce inflammation	Restore nerve mobility and reduce perineural adhesions while delivering a therapeutic injectate
Typical injectate volume	Approximately 1–3 mL	Approximately 5–15 mL
Onset of symptom relief	Often within days to weeks	Typically within days to weeks
Evidence base	Extensive; supported by multiple randomized controlled trials, systematic reviews, and clinical practice guidelines	Growing; supported by randomized controlled trials, systematic reviews, and network meta-analyses
Short-term efficacy	Well-established improvement in pain, paresthesias, and functional outcomes	Demonstrated improvement in pain, symptom severity, and functional outcomes
Long-term durability	Variable; symptom recurrence is common over time	Emerging evidence suggests potentially longer-lasting symptom improvement in some patients
Effect on mechanical nerve entrapment	Primarily indirect through reduction of inflammation and edema	Directly addresses perineural adhesions and impaired nerve gliding through hydrodissection
Hyperglycemia risk	Possible transient elevation in blood glucose, particularly in patients with diabetes mellitus	No known clinically significant effect on blood glucose levels
Skin hypopigmentation or subcutaneous fat atrophy	Rare but recognized complications	Not reported in the current CTS literature
Repeat treatment considerations	Repeated injections may be limited by cumulative corticosteroid exposure	May be repeated without concerns related to corticosteroid exposure
Technical complexity	Relatively straightforward ultrasound-guided procedure	More technically variable; procedural complexity depends on needle approach, target tissue plane, and degree of circumferential nerve separation
Current clinical role	Established first-line injectable therapy for mild-to-moderate CTS	Emerging alternative or adjunctive therapy, particularly for patients seeking to avoid corticosteroids
Major limitations	Benefits may diminish over time; does not directly address perineural adhesions or impaired nerve mobility	Variability in hydrodissection technique, injectate volume, target tissue planes, procedural endpoints, and long-term comparative evidence

Direct comparative studies have demonstrated that both interventions produce clinically meaningful improvements in pain, symptom severity, and functional outcomes. However, several comparative investigations suggest that D5W hydrodissection may provide more durable symptom improvement at intermediate- and long-term follow-up in selected patients. In a randomized double-blind trial comparing D5W with triamcinolone injection, Wu et al. demonstrated greater improvements in symptom severity and functional outcomes with D5W at later follow-up intervals despite similar early clinical responses [17]. These findings support the hypothesis that mechanical release of perineural adhesions may provide therapeutic benefits beyond those achieved through anti-inflammatory effects alone [2,7].

Despite these encouraging findings, current evidence does not conclusively establish the superiority of D5W hydrodissection over CSI in all patient populations. Considerable heterogeneity exists among published studies with respect to hydrodissection technique, injectate volume, target tissue planes, degree of circumferential median nerve release, treatment frequency, outcome measures, CTS severity, and duration of follow-up [2,14]. These procedural differences may influence clinical outcomes independently of injectate selection and should be considered when interpreting comparative studies. Consequently, interpretation of the available literature should be undertaken with caution.

At present, CSI remains the most established first-line injectable therapy for CTS because of its extensive evidence base, widespread availability, predictable short-term efficacy, and strong support from current clinical practice guidelines [5,6,9,12]. D5W hydrodissection represents a promising alternative for appropriately selected patients and may provide more durable symptom improvement while avoiding corticosteroid-related adverse effects. Nevertheless, additional multicenter randomized controlled trials with standardized hydrodissection techniques, injectate volumes, treatment protocols, and longer follow-up periods are needed to better define the relative roles of these interventions and determine which patients are most likely to benefit from each approach [2,9,14,17,19].

Limitations of current evidence

Although the available literature evaluating D5W hydrodissection for CTS is encouraging, several important limitations warrant consideration. First, the overall body of evidence remains substantially smaller than that supporting CSI. Many studies evaluating D5W hydrodissection involve relatively small sample sizes and originate from a limited number of investigative groups, which may affect the generalizability of findings across broader patient populations. Furthermore, many available studies have been conducted at single centers, underscoring the need for larger multicenter investigations.

Significant heterogeneity also exists among published hydrodissection studies. Variations in needle approach, target tissue planes, injectate volume, degree of circumferential median nerve separation, number of treatment sessions, outcome measures, and duration of follow-up make direct comparisons between studies challenging. Furthermore, some investigations evaluate perineural D5W injection without true hydrodissection, whereas others employ varying degrees of circumferential nerve release, introducing additional methodological variability. Although corticosteroid injection protocols are generally more standardized, differences in corticosteroid preparation, dose, injectate composition, and the use of local anesthetic also contribute to variability across comparative studies [2,7,15,16].

Long-term comparative data remain limited. Although several studies suggest that D5W hydrodissection may provide more durable symptom improvement than CSI in selected patients, relatively few large multicenter randomized controlled trials have directly compared these interventions. Consequently, definitive conclusions regarding the comparative effectiveness of these treatments cannot currently be made, and existing findings should be interpreted within the context of considerable methodological heterogeneity.

Future research should focus on multicenter randomized controlled trials employing standardized procedural protocols, including needle approach, target tissue planes, injectate volume, degree of circumferential nerve release, treatment frequency, corticosteroid preparation, and outcome measures. Standardization of these variables will improve study comparability, facilitate more robust evidence synthesis, and help define optimal patient selection while further clarifying the relative roles of CSI and D5W hydrodissection in the nonoperative management of CTS [2,7,14].

Clinical implications

Current evidence supports ultrasound-guided CSI as the preferred first-line injectable intervention for patients with mild-to-moderate CTS who fail initial conservative treatment. Mild-to-moderate CTS generally refers to patients without thenar muscle atrophy, significant motor weakness, or severe electrodiagnostic abnormalities who continue to experience bothersome symptoms despite splinting, activity modification, and other conservative measures. CSI is supported by a robust body of evidence demonstrating reliable short-term improvements in pain, paresthesias, and functional outcomes, making it the benchmark against which emerging injectable therapies are compared [5,6,9,12].

D5W hydrodissection may represent a reasonable alternative or adjunctive treatment option in selected patients. Potential candidates include individuals with poorly controlled diabetes mellitus, patients wishing to avoid corticosteroid exposure, those who have experienced symptom recurrence following CSI, or patients suspected of having significant perineural adhesions contributing to persistent nerve entrapment. Although current evidence is encouraging, D5W hydrodissection should not be viewed as a replacement for CSI at present. Rather, it should be considered an emerging treatment option whose use should be individualized based on patient characteristics, provider experience, and the available evidence. In addition to injectate selection, procedural success likely depends on appropriate execution of the hydrodissection technique, including accurate needle placement, adequate separation of the median nerve from surrounding tissues, and familiarity with ultrasound-guided peripheral nerve interventions [2,7].

Patients with severe CTS characterized by thenar atrophy, persistent sensory loss, significant motor weakness, severe electrodiagnostic abnormalities, or progressive neurologic dysfunction should be referred for surgical evaluation, as these findings suggest advanced median nerve compression that is less likely to respond to injection therapy alone. Surgical decompression remains the definitive treatment for advanced CTS and should not be unnecessarily delayed in patients with clear indications for operative management [9,12].

As additional comparative evidence becomes available, the role of D5W hydrodissection within the CTS treatment algorithm will likely become better defined. Future studies employing standardized hydrodissection techniques and long-term follow-up will help clarify which patients are most likely to benefit from D5W hydrodissection, either as an alternative to or in combination with established treatment strategies [2,14,17,19].

## Conclusions

Ultrasound-guided perineural CSI and D5W hydrodissection are both effective nonoperative treatment options for CTS. CSI remains the most established injectable therapy and is supported by a robust body of evidence demonstrating reliable short-term improvements in pain, symptom severity, and functional outcomes. Its widespread availability, favorable safety profile, and strong guideline support continue to support its role as the preferred first-line injectable intervention for patients with mild-to-moderate CTS. D5W hydrodissection has emerged as a promising alternative that may provide additional therapeutic benefits through mechanical release of perineural adhesions, restoration of median nerve mobility, and modulation of neurogenic inflammation. Current evidence suggests that D5W hydrodissection may provide more durable symptom improvement in selected patients while avoiding corticosteroid-related adverse effects. However, available comparative studies remain heterogeneous, and the current evidence is insufficient to establish definitive superiority over CSI. Although the available evidence is encouraging, important variability persists among published studies with respect to hydrodissection technique, injectate volume, treatment protocols, outcome measures, and duration of follow-up. Future multicenter randomized controlled trials employing standardized procedural protocols and longer follow-up periods are needed to better define comparative effectiveness, optimize patient selection, and clarify the role of D5W hydrodissection within the CTS treatment algorithm. Until higher-quality comparative evidence becomes available, treatment selection should remain individualized, with consideration of patient characteristics, clinician expertise, and the current evidence base.
